# Pioglitazone Treatment Increases Survival and Prevents Body Weight Loss in Tumor–Bearing Animals: Possible Anti-Cachectic Effect

**DOI:** 10.1371/journal.pone.0122660

**Published:** 2015-03-25

**Authors:** Mércia Beluzi, Sidney B. Peres, Felipe S. Henriques, Rogério A. L. Sertié, Felipe O. Franco, Kaltinaitis B. Santos, Pâmela Knobl, Sandra Andreotti, Cláudio S. Shida, Rodrigo X. Neves, Stephen R. Farmer, Marília Seelaender, Fábio B. Lima, Miguel L. Batista Jr.

**Affiliations:** 1 Laboratory of Adipose Tissue Biology, Integrated Group of Biotechnology, University of Mogi das Cruzes, Mogi das Cruzes, Brazil; 2 Cancer Metabolism Research Group, Institute of Biomedical Sciences, University of Sao Paulo, São Paulo, Brazil; 3 Laboratory of Physiology, Institute of Biomedical Sciences, University of Sao Paulo, São Paulo, Brazil; 4 Department of Physiological Sciences, State University of Maringá, Paraná, Brazil; 5 Department of Biomedical Engineering, Federal University of Sao Paulo, Sao Jose dos Campos, Brazil; 6 Department of Biochemistry, Boston School of Medicine, Boston, Massachusetts, United States of America; National Institute of Agronomic Research, FRANCE

## Abstract

Cachexia is a multifactorial syndrome characterized by profound involuntary weight loss, fat depletion, skeletal muscle wasting, and asthenia; all symptoms are not entirely attributable to inadequate nutritional intake. Adipose tissue and skeletal muscle loss during cancer cachexia development has been described systematically. The former was proposed to precede and be more rapid than the latter, which presents a means for the early detection of cachexia in cancer patients. Recently, pioglitazone (PGZ) was proposed to exhibit anti-cancer properties, including a reduction in insulin resistance and adipose tissue loss; nevertheless, few studies have evaluated its effect on survival. For greater insight into a potential anti-cachectic effect due to PGZ, 8-week-old male Wistar rats were subcutaneously inoculated with 1 mL (2×10^7^) of Walker 256 tumor cells. The animals were randomly assigned to two experimental groups: TC (tumor + saline-control) and TP5 (tumor + PGZ/5 mg). Body weight, food ingestion and tumor growth were measured at baseline and after removal of tumor on days 7, 14 and 26. Samples from different visceral adipose tissue (AT) depots were collected on days 7 and 14 and stored at -80o C (5 to 7 animals per day/group). The PGZ treatment showed an increase in the survival average of 27.3% (P< 0.01) when compared to TC. It was also associated with enhanced body mass preservation (40.7 and 56.3%, p< 0.01) on day 14 and 26 compared with the TC group. The treatment also reduced the final tumor mass (53.4%, p<0.05) and anorexia compared with the TC group during late-stage cachexia. The retroperitoneal AT (RPAT) mass was preserved on day 7 compared with the TC group during the same experimental period. Such effect also demonstrates inverse relationship with tumor growth, on day 14. Gene expression of PPAR-γ, adiponectin, LPL and C/EBP-α from cachectic rats was upregulated after PGZ. Glucose uptake from adipocyte cells (RPAT) was entirely re-established due to PGZ treatment. Taken together, the results demonstrate beneficial effects of PGZ treatment at both the early and final stages of cachexia.

## Introduction

Cachexia is a multifactorial syndrome characterized by profound involuntary weight loss, fat depletion, skeletal muscle wasting, and asthenia; all symptoms are not solely attributable to inadequate nutritional intake [1,2]. Cachexia is implicated in up to 40% of all cancer deaths and is directly responsible for an impaired quality of life and increased health care costs [3]. Clinical treatments to overcome cachexia and its outcomes are currently unavailable.

Loss of both adipose tissue and skeletal muscle mass during cancer cachexia development has been well-characterized and systematically described [4,5]. The former is proposed to precede and occur more rapidly than the latter [6]. For this aspect, several factors have been proposed to cause adipose tissue loss and metabolic “chaos”, such as increased lipid mobilization due to enhanced adipocyte lipolysis [5,7], reduced lipogenesis due to decreased lipoprotein lipase activity [8], down-regulation of key adipogenic factors [9,10] and insulin resistance [11]. We previously showed that some of these changes occur at a very early stage during cancer cachexia development, even before the clinical signs of cachexia appear in tumor-bearing rats. The rats exhibited substantially reduced gene transcription and protein expression of crucial adipogenic proteins, such as PPAR-γ, C/EBPα and perilipin in visceral depots during the early stages of cachexia. The disruption was followed by adipocyte morphological modifications, which became progressively more evident during the late disease stages [10].

Thiazolidinedione (TZD) encompasses a class of drugs that activate peroxisome proliferator activated receptor gamma (PPARγ), which is a key transcription factor that induces insulin-sensitive genes and is targeted in Type II diabetes mellitus treatment [12,13]. In recent studies, this drug class exhibited anti-cancer properties; Troglitazone (TGZ) inhibits colorectal, breast, and prostate cancer cell growth [14,15], and pioglitazone (PGZ) arrests the cell cycle and induces primary liposarcoma cell differentiation [16,17]. A reduced risk of lung cancer has also been reported [18]. Despite these encouraging results, associated toxicities inhibit clinical therapeutic potential [19].

In addition to its anti-cancer effect, in recent studies with cachectic mice bearing colon-26 tumors, rosiglitazone treatment improved insulin sensitivity and attenuated skeletal muscle protein degradation during early cachexia [11,20]. However, during the experimental period, the tumor mass was unaffected by the treatment. Furthermore, the final body weight was not correlated to tumor size, which indicates that the effects of rosiglitazone in attenuating cachexia did not depend on tumor size. On the other hand, despite a previous study that showed an initial anti-cachectic effect, cachexia development was only evaluated over a 14-day period, which may be an insufficient timeframe for evaluating its effectiveness on body weight loss and tumor growth. In addition, no PGZ dose-response curves are available for cachexia markers.

For greater insight into a potential anti-cachexia effect from PGZ during cancer cachexia development, we analyzed the RPAT and MEAT depots at different time points following tumor injection and PGZ treatment. Herein, we present the following results: 1) a TZD dose-dependent response that affects survival; 2) body weight and tumor mass evolution during cachexia progress; and 3) insulin-induced glucose uptake from isolated adipocytes at the early and final stages of cachexia. Considering each factor, the results show that both body and tumor mass are preserved during the final cachexia stages, which correlates with the PGZ dose-concentration. In addition, the results suggest that the retroperitoneal adipose pad (RPAT), which is most affected in Walker tumor-bearing animals, is more responsive to PGZ when the treatment begins upon onset of cachexia.

## Material and Methods

### Animals

Male adult Wistar rats (160–250 g), obtained from the Institute of Biomedical Sciences, University of São Paulo, were maintained in metabolic cages, in a 12 h light: 12 h dark cycle (lights on at 07:00 h), under controlled temperature conditions (23±1°C), receiving water and food (NuvilabCR1-Nuvital, Curitiba, Paraná, Brazil), ad libitum. The Ethical Committee for Animal Research from the University of Mogi das Cruzes approved all the adopted procedures, which were carried out in accordance with the ethical principles stated by the Brazilian College of Animal Experimentation (BCAA)—Protocol n. 008/2011.

### Experimental Design

#### Study 1

In order to determine an initial dosage of PGZ capable of inducing modification on survival rate, a pilot study was conducted. Therefore, we tested four doses of the drug adjusted to rat body weight (mg / kg body weight / day) at the following concentrations; 0 (NaCl 0.9%), 5, 10, 20 and 40 mg/kg/day, which was administered orally by intragastric gavage. For all groups, PGZ or saline administration were carried out throughout the same vehicle solution, sodium chloride 0.9% plus 0,1% DMSO. The treatment was administered daily from one week before inoculation of the tumor cells up to the 28^th^ day. The experimental groups were divided according to dose-treatment: TC (tumor + saline-control), TP5 (tumor + PGZ/5mg), TP10 (tumour + PGZ/10mg), TP20 (tumor + PGZ/20mg), TP40 (tumor + PGZ/40mg).

Weight and food intake were assessed daily, always in the afternoon. Walker 256 tumor cells (2 x10^7^ cells) were injected subcutaneously (s.c.) into the right flank of the animals [21,22]. Control rats received saline injections on the same day of tumor inoculation. Cachexia is usually observed in rats bearing Walker 256 tumors after 10–15 days [23]. To evaluate the effect of PGZ treatment during cancer cachexia progression, the experiments were carried out as a time course study. The impact of tumor growth and development of cachexia on animal welfare were considered, with appropriate measures to monitor and alleviate suffering implementation. Rats were euthanized by overdose of anesthetic (thiopental sodium) injection at 28 days, or when weight loss reached 35%, or tumor size reached 65 mm, whichever occurred first.

#### Study 2

After the initial characterization considering the lowest dose capable of inducing increased animal survival, male Wistar rats were randomly assigned to two experimental groups, as follows; TC (tumor + saline-control) and TP5 (tumor + PGZ/5mg). PGZ suspension was prepared as described (Study 1). Walker 256 tumor cells (2x10^7^ cells) were subcutaneously injected into the right flank of the animals [21,22]. Control rats received saline injections on the same day of tumor cells inoculation. To evaluate PGZ treatment (5 mg) effect during cancer cachexia progression, in particular considering clinical signs (body weight, food ingestion and tumor growth) the experiments were carried out as a time course study, considering three different experimental time points (7, 14 or 26 days post-injection). Such periods were chosen considering identification of the early and end points of the cachexia trajectory [10]. To evaluate the impact of cachexia in WAT depots, experiments were carried out at two different experimental times (7 and 14 days post-injection). Rats were euthanized on days 7, 14 or 26 post-injection (seven to ten animals on each time point) with thiopental sodium (50 μg/g body wt) injection followed by decapitation after 12 h fasting. For the biochemical analysis, an additional control group was included to access baseline values.

### Blood sampling and adipose tissue collection

Trunk blood was collected after decapitation into 15 ml conical tubes containing EDTA (1.8 mg/ml of blood), centrifuged at 500 g and 4°C for 10 min, and stored at **-**80°C. Epididymal (EAT), retroperitoneal (RPAT) and mesenteric (MEAT) adipose tissue depots (after careful removal of adjacent lymph nodes) were removed, weighed, snap frozen in liquid nitrogen and stored at **-**80°C.

### General Biochemical parameters

The concentration of triacylglycerol, glucose, albumin, and the activity of liver enzymes: aspartate aminotransferase (AST), alanine aminotransferase (ALT), and total protein were measured by commercial kits (Labtest, Brazil). For general biochemical analysis, it was used a control baseline group, matched by age, with neither tumor cells, neither saline inoculation, nor PGZ treatment.

### Adipocyte isolation

Adipocytes were isolated by collagenase treatment of adipose tissue as previously described [24]. The cell suspensions (40% final concentration corresponding to 10^6^ cells/ml) were incubated in a 37°C water bath for 30 min before initiating the biological tests as detailed below. Adipocyte viability and number were determined as previously described [25].

### RT-qPCR

Total RNA was isolated from isolated adipocytes from the RPAT samples employing Trizol. (Invitrogen, Carlsbad, CA, USA) following the manufacturer’s recommendations. The concentration of the RNA extract was determined using the Synergy H1 Multi-Mode Microplate Reader (BioTek Instruments, Winooski, VT, USA) with the Take3 microdrop addition at an absorbance of 260 nm (A_260_) and 280 nm (A_280_) in 10 mM Tris-Cl at pH 7.5. The estimative of RNA purity was determined by calculating the ratio between the two readings (A_260_/A_280_). RNA integrity was checked on a 2% agarose gel containing SYBR Safe DNA gel stain (Invitrogen Corporation, Carlsbad, CA, USA). (Invitrogen Corporation, Carlsbad, CA, USA). RT-PCR was performed on the total RNA (4 μg), which was employed for first-strand synthesis of cDNA using a commercially available kit (Ambion, Austin, TX, USA). The reaction mixture was stored at -80°C until the PCR step. Primer sets for rat PPAR-γ (NM 001145366.1 sense 5`-CTG CCT ATG AGC ACT TCA CAA-3 and antisense 5`-CGT TGG CTGCGT TCA TGT CGT AAT-3`), C/EBP-α (NM 012524.2: sense, 5`-GAA GTC GGT GGA TAA GAA CAG C-3`and antisense, 5`-GGT CAT TGT CAC TGG TCA ACT C-3`), SREBP-1c (XM 213329.5:sense, 5`-AGG GAG TTC TCA GAT GCT CTT G-3 and antisense, 5`-CAT GCT GGA ACT GAC AGA GAA G-3`), adiponectin (NM 144744.3: sense, 5`-ATC CTG CCC AGT CAT GAA GGG ATT-3`and antisense, 5`-TGC CAT CCA ACC TGC ACA AGT TTC-3`), LPL (NM 012598.2: sense, 5`-GAG TTT GGC TCC AGA GTT TGA-3`and antisense, 5`-GTG TCC TCA GCT GTG TCT TCA-3`), FAS (NM 139194.2: sense, 5`-CAT TTT GCT GTC AAC CGT GT-3`and antisense, 5`-CGT GTA CTC CTC CCC TTC TG-3`), were designed using Primer Express software v2.0 (Applied Biosystems, Foster City, CA, USA). The results for mRNA concentrations are expressed as a ratio over 18S-ribosomal ribonucleic acid (rRNA), which was amplified as a housekeeping gene using the following primers: 18S rRNA (M11188.1 sense 5`-TCA GCT TTG CAA CCA TAC TCC -3`, and antisense 5`-GAC CAT AAA CGA TGC CGA CT-3`). For each sample, PCR was performed in duplicate in a 25-μL reaction volume of 5–20 ng of cDNA, 12.5 μL Syber Green Master Mix (Applied Biosystems), and 200–600 nM of each primer. PCR analyses were carried out using the following cycle parameters: 50°C for 2 min, 95°C for 10 min, followed by 40 cycles of 95°C for 15 s and 60°C for 1 min. Fluorescence was quantified and analyses of amplification plots were performed with the ABI Prism 7700 Sequence Detector System (Applied Biosystems, Foster City, CA). All samples were normalized to the 18S values and the results were expressed as fold changes of Ct values relative to controls adopting the 2^–DDCt^ formula. 18S was adopted as a reference gene, since we have experimentally determined that there were no statistically significant differences of its Ct values between control and different times of the tumor-bearing groups (both P<0.05, three experiments).

### [^3^H]-2DG uptake assay

#### Deoxy-D[2,6 3H]glucose uptake (2DGU) in isolated adipocytes

2DGU experiments were performed as described elsewhere [26] with some modifications. Briefly, aliquots (40 μL) of isolated adipocytes (40–50% cell suspension) were transferred to 2-ml plastic test tubes with or without insulin (2.5 nmol/L) diluted in EHB buffer 2 (pH 7.4), and the cells were incubated for 15 min in a water bath at 37°C. At the end of the incubation period, a 10-μl aliquot of 2-deoxy-D-[^3^H]-glucose was added (^3^H-2DG, 0.4 mmol/l final concentration and 0.05 μCi/tube) and the uptake reaction was allowed to occur for exactly 3 min. The reaction was interrupted by adding 250 μL of ice-cold phloretin (0.6 mmol/l in EHB and DMSO 0.05%). Next, 200 μL of this last mixture were transferred to microfuge tubes (450-μl capacity), layered with 200 μL of silicone oil (density = 0.963 mg/ml) and centrifuged (Microfuge E, Beckman Instruments, Palo Alto, CA) for 10 s at 11.000 *g*. The cell pellet on top of the oil layer was transferred to 4-mL vials containing 2.5 mL of scintillation cocktail (EcoLume, ICN Pharmaceuticals, Costa Mesa, CA, USA), and the trapped radioactivity was measured in a liquid scintillation counter (MicroBetaTriLux 1450 LSC & luminescence Counter, PerkinElmer, USA). Unspecific ^3^H-2DG radiolabel trapping was determined in a parallel tube already prepared with 250 μl of ice-cold phloretin to stop transport reaction before adding the tracer. This value was discounted from the total trapping, and the resultant specific uptake was recalculated and expressed as picomoles (pmol) per square centimeter of cell surface area.

### Statistical analysis

Data are expressed as mean values ± S.E.M. Differences between times of tumor cell injection (cachexia progression: days 0, 7, 14 and 26) were analyzed by one-way ANOVA followed by Bonferroni’s post-hoc comparisons tests using GRAPHPAD PRISM software for Macintosh, version 6.0 (GraphPad, San Diego, CA, USA). The differences between qualitative variables on days 7 and 14 against control (matched by age) were determined by the unpaired form of the Student’s t-test. Kaplan-Meier survival test was performed to evaluate animal survival, using the same software. We calculated the correlation coefficient between the measures employing the nonparametric Spearman correlation statistic (*r*) and the correlation values were calculated with 2-tailed significance. Glucose transport (2DGU) studies were analyzed with one-way ANOVA followed by Bonferroni’s post-hoc comparisons. P<0.05 was considered significant for all statistical tests.

## Results

### Study 1

#### Determining the most efficient PZD dosage

**Fig 1:**
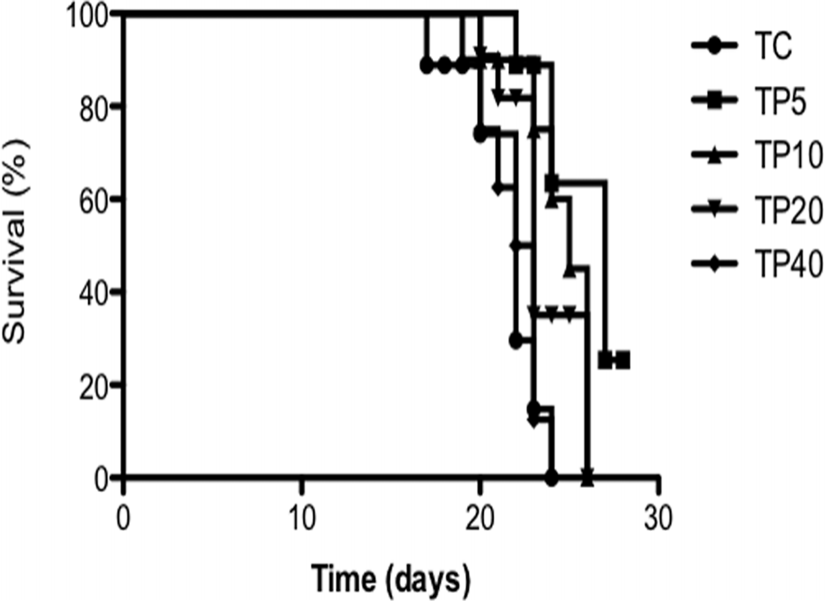
Survival of tumor-bearing animals during PGZ treatment period. To evaluate dose-response drug effect, animals were treated with four doses of PGZ normalized by rat’s body weight, which were administered daily: 5, 10, 20 and 40 mg/kg/day. Plots of Kaplan-Mayer product limit estimates of survival of a group of tumor-bearing animals receiving PGZ therapy during 26 days, ^#^ p<0.05 vs. TC.

To evaluate the most efficient PGZ dosage for prolonging the survival of tumor-bearing rats, we tested four different doses of the drug normalized by animal body weight, administered daily: 5, 10, 20 and 40 mg/kg/day (Study 1). During the period of treatment (28 days), the first death occurred in an animal of the control group (TC), on day 17 after the inoculation of tumor cells. At the other extreme of the experiment, 36.3% of animals treated with 5 mg of PGZ (TP5) were still alive on day 28 of the experiment. The rat’s life span is shown in Fig. 1.

The animals in TP5 group showed a survival average of 27 days. This represents an increase of 27.3% (P< 0.01) when compared to TC, which showed a survival median of 21 days. In the other groups survival was inversely correlated with dosage, 25 (TP10), 23 (TP10) and 22.5 (TP40) days—no difference to the control group (TC). We thereafter preceded to the second phase of the study, adopting the minimal dose to affecting survival, 5 mg/kg/day (Study 2).

#### Characterization of Total Body Mass, tumor growth and food intake in Rats

**Fig 2:**
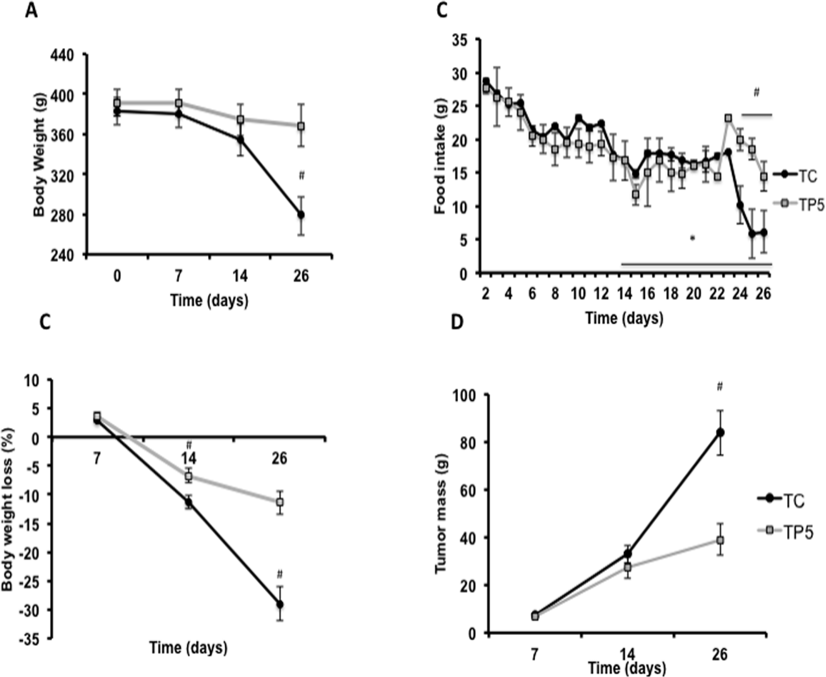
Body weight and food intake for different time points along the progression of cachexia. Rats were inoculated with Walker 256 tumor cells (2×10^7^ cells) or vehicle and treated daily with 5 mg of PGZ (TP5) or PBS (TC) during 26 days. (A) Body weight was measured on days 4, 7, 14 and 26 and (B) Relative body weight loss on days 7, 14 and 26-post tumor cells injection. (C) Food intake was measured daily throughout the study and (D) Tumor mass was measured on days 7, 14 and 26-post tumor cells inoculation. * p<0.05, one-way ANOVA followed by Bonferroni’s post-hoc (time from day 0), and ^#^ P<0.05 vs. TC.

As body weight loss is the hallmark of cachexia, this parameter was observed daily along the entire experimental period, comprising 26 days, as shown in Fig. 2.

This is an important parameter, enabling classification of cachexia stages. Body weight loss had to be greater than 5% of total body mass [1] in order to characterize the cachectic state. TP5 group showed a slighter reduction of the percentage of body mass of 4.5% (day 14) and 5.9% (day 26, p< 0.05 for both), compared to the initial experimental period (day 0). TC showed a reduction of about 11.3% on day 14 and 26.9% on day 26 (P<0.01 for both), being that the greater period of body mass reduction when comparing to TP5 group (40.7, day 14 and 56.3%, for day 26, p<0.01), during the same experimental period (Fig. 2A and B). Another important cachexia-associated symptom is anorexia. In our experimental model of cachexia-induced by Walker 256 tumor-bearing rats, diminished appetite could be detected between the 10^th^ and 14^th^ day after cells inoculation [10] (Fig. 2C). The animals of both groups (TC and TP5) presented a reduction of food intake beginning on day 13 (39% and 38%, p<0.05 respectively), as compared to the initial experimental period. However, from day 23, PGZ treatment was able to attenuate anorexia, as TP5 showed the reduction in food intake of 38% (P<0.01), while in TC showed a decrease of 64% (P<0.001), both measured on the 26^th^ day after tumor cells inoculation (Fig. 2C). We also measured tumor mass on days 7, 14, and 26 (Fig. 2D). The tumor was dissected and weighed on days 7, 14 and 26 post-injection. No significant difference was detected on days 7 and 14, considering the relative weight and treatment among rats. On day 26 there was a reduction of tumor growth in TP5 (53.4%, p<0.01), as compared to TC (Fig. 2D).

### Study 2

#### Characterization of early PGZ effect on AT mass during cachexia

**Fig 3:**
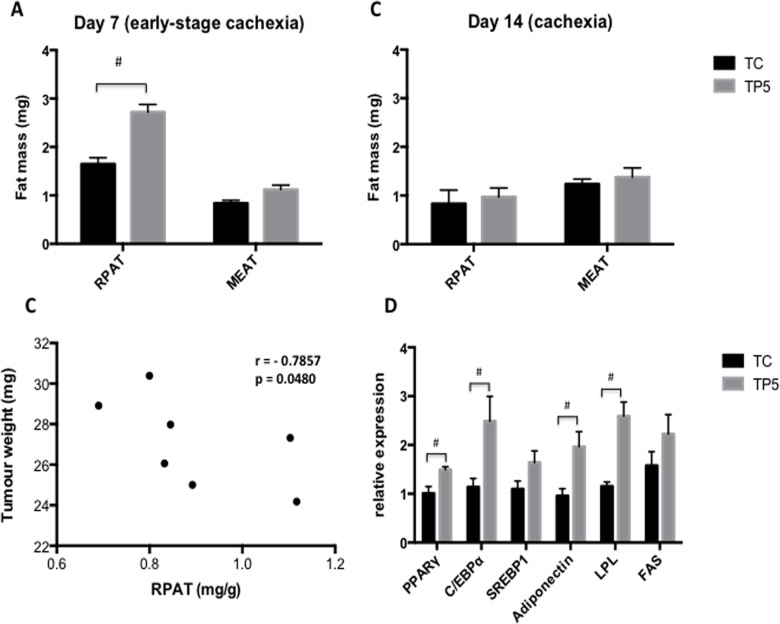
Visceral adipose tissue mass during the progression of cachexia. Rats were inoculated with Walker 256 tumor cells (2×10^7^ cells) or vehicle and treated daily with 5 mg of PGZ (TP5) or PBS (TC) during 14 days. (A) RPAT and (C) MEAT tissues from tumor-bearing rats were removed and weighed on day 7 and day 14. (B) Spearman correlation between relative RPAT mass on day 7 (early-stage) and tumor growth on day 14 (cachexia-stage), from TP5. (D) Real-time PCR analysis of RNA isolated from RPAT (isolated adipocytes) in the 7th on day after tumor cell inoculation. mRNA levels of target genes were normalized to 18S. Values are mean ± s.e.m. for five to seven animals per group. RPAT-retroperitoneal adipose tissue; MEAT-Mesenteric adipose tissue. ^#^p<0.05 vs. TC.

After euthanasia, the tissues were collected from retroperitoneal (RPAT) and mesenteric (MEAT) fat depots and then weighed on the 7^th^ and 14^th^ days after inoculation of tumor cells, to evaluate the treatment effect on the early signs of cachexia (day 7) and day 14, when the clinical signs of cachexia are already established (Fig. 3).

RPAT from TP5 was preserved on day 7, showing higher values (25.5%, p<0.05) when compared to TC in the same experimental period. On the 14^th^ day there was no difference between TC and TP5. In addition, there was no difference between these groups regarding MEAT during the same time span (Fig. 3A and C). After we detected a transient and precocious preservation of RPAT mass, we evaluated a possible correlation considering PGZ effect on RPAT mass (day 7) and reduced tumor mass growth, found in TP5 on day 14. A negative correlation was present in relation on higher RPAT mass and lower tumor growth (*r* = -0.7857, p<0.05) (Fig. 3B). To gain additional insight regarding the PGZ effect in the early stage of cachexia, we examined, in isolated adipocytes from RAPT depots, the expression of genes regulated by PPAR agonists, such PPAR-γ, Adiponectin and lipoprotein lipase (LPL), in RPAT from cachectic rats. We also examined major transcription factors involved in fat cell formation and maturation, C/EBP-α, SREBP-1c and fat acid synthase (FAS) (Fig. 3D). Gene expression of PPAR-γ, adiponectin and LPL from TP5 adipocytes showed higher levels on day 7, increasing 48.5% (p<0.01), 1.1 and 1.2-fold (p<0.001, for both), respectively, when compared with TC samples. C/EBP-α expression was also increased after PGZ treatment, showing 1.2-fold increase, when compared with TC, on day 7. SREBP-1c and FAS gene expression was not affected by the treatment.

#### Biochemical Tests

Plasma and serum biochemical parameters of rats treated with PGZ (TP5), vehicle solution (TC) and of the control group were determined on days 7 and 14 after inoculation of tumor cells (Table 1).

**Table 1:** Biochemical parameters from tumor-bearing animals during PGZ treatment.

				day 7						day 14					
Biochemical parameters	Control			TP5			TC			TP5			TC			
Glucose (mg/L)	172	±	3.8	105	±	4.8 ^&^ ^#^	84	±	6.0^&^	115	±	5.0^&^ ^#^ *	72	±	5.3^&^	
TAG (mg/dL)	64	±	5.6	71	±	3.9	76	±	1.5	211	±	14^&^ ^#^ *	180	±	3.0^&^ ^#^	
AST (U/mL)	6.2	±	2.2	14	±	1.7^&^	15	±	2.7^&^	31	±	3.1^&^ ^#^ *	13	±	1.1^&^	
ALT (U/mL)	7.4	±	3.3	6.1	±	2.7	6.3	±	3.7	16	±	6.5^&^	11	±	6.2	
Albumin (g/dL)	2.9	±	0.1	1.9	±	0.1^&^	1.9	±	0.1^&^	1.8	±	0.1^&^	2.1	±	0.1^&^	

Values are mean **±**s.e.m. for five to seven animals per group.

* p<0.05, one-way ANOVA followed by Bonferroni’s post-hoc (day 7 vs. 14)

^#^ P<0.05 vs. TC,

^&^ P<0.01 vs. Baseline Control.

The biochemical profile of animals, either treated with the drug (TP5) or with vehicle (TC) showed significant alteration in the following parameters. Cachexia-induced hypoglycemia was detected in TC both on day 7 and 14 (51.3 and 58.4%, respectively), in comparison to the control group. PGZ treatment, however, attenuated the decrease of fasting blood glucose levels by 12.0% (p<0.01), when compared with TC, on day 7. Treatment (TP5) showed to keep attenuation effect of hypoglycemia induced by the presence of the tumor, and one such effect was even more pronounced (25.1%, p<0.001) compared to TC, on day 14. Circulating TAG levels showed no alteration on day 7 for both groups (TP5 and TC), while a still higher concentration on day 14 (2.3 and 1.8-fold, respectively, p<0.001) in relation to control was found. Additionally, the hepatic enzymes evaluated were also changed. AST displayed a gradual increase in TP5 in relation to the control group, 1.2-fold on day 7 and 3.9-fold on day 14 (p<0.001, respectively). For TC, the increase was of 14.1% on day 7 and 11.6% on day 14 (p<0.01, respectively). On day 14, ALT also showed a marked increase in TP5 when compared to the control group (49.5%, p<0.05). Albuminemia was lower in TP5 and TC on day 7 (34.4%, p<0.05), when compared to the control group. On day 14, the albumin levels were 36.8%, (p<0.05) lower in TP5 and 26.5% (p<0.05) in TC, compared to the control group on the same day.

#### (3H)-2DG uptake in isolated adipocytes

**Fig 4:**
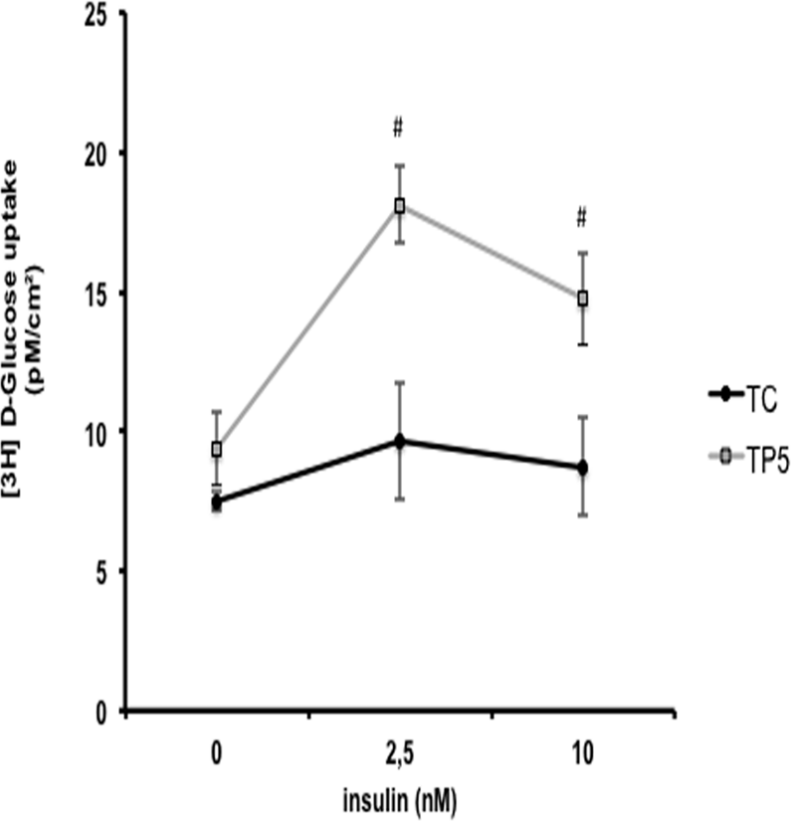
2-Deoxy-D[2,6 ^3^H]glucose uptake assay. Rats were inoculated with Walker 256 tumor cells (2×10^7^ cells) or vehicle and treated daily with 5 mg of PGZ (TP5) or PBS (TC) during 7 days. Isolated adipocytes from RPAT tissues were stimulated with 0 (basal), 2.5 and 10 (maximal) nmol/cm^2^ of cell surface area. RPAT-retroperitoneal adipose tissue; * p<0.05, one-way ANOVA followed by Bonferroni’s post-hoc (time from day 0), ^#^ P<0.05 vs. TC, and ^&^ P<0.05 vs. Baseline Control.

In the attempt to evaluate the interaction between cachexia and PGZ treatment on cell sensitivity to insulin, a glucose uptake test was done using isolated adipose cells from RPAT depot. This AT depot was chosen considering that it is the most affected by the syndrome. We evaluated this parameter on day 7 after the inoculation of tumor cells, since this was the period in which the treatment efficiently inhibited the reduction in RPAT mass induced by cachexia. Cells were stimulated by a gradient of increasing insulin concentration (0, 2.5 e 10 nM). The results express tracer flow through the cell surface area (Fig. 4).

TP5 cells showed higher 2DG uptake (93.2%, p<0.005) when stimulated with 2.5 nM of insulin, compared with TC, on day 7. When stimulated with 10 nM of insulin, in the same experimental period, adipocytes from TP5 also showed higher 2DG uptake (57.1%, p<0.01) compared with TC. Treatment with TP5 totally reverted the effect of cachexia on 2DG uptake by RPAT adipocytes, that showed no response to insulin ranging from basal to maximal (10 nM) stimulation.

## Discussion

We previously demonstrated [10] that, even before classical cachexia symptoms appear, AT mass reduction, which occurs before lean mass loss, may be associated with the down-regulation of adipogenic and lipogenic genes during the early stages of the disease. In addition, several studies have recognized the importance of triglyceride hydrolysis as a major metabolic pathway involved in cancer cachexia initiation and/or progression [5,27]. Thus, considering the well-described adipogenic and lipogenic effect of PGZ treatment, we initially tested a hypothesis that pharmacological intervention with PGZ prevents AT loss, at least during the early stages of cachexia. Consequently, cancer cachexia outcome improves, suggesting this as a possible clinical tool for improving quality of life in patients who develop the syndrome. The foremost finding of the present study is that PGZ prolongs survival and preserves visceral fat, notably in the RPAT depot, mainly during the early phases of the disease (pre-cachexia). Body mass loss and tumor growth were also attenuated when cachexia signs were present (late-stage to refractory cachexia). Additionally, transitive and early preservation of RPAT mass seems related with the preservation of insulin-stimulated glucose-uptake mechanisms, which is a direct effect of PGZ treatment.

### 4.1. Determining the ideal dosage and main effects on the clinical signs of cachexia

The literature includes many collateral effects associated with the use of TZDs. The most commonly described effect is hepatotoxicity, which is related to the dose and type of TZD. Nonetheless, changes such as retention of fluid, cardiac hypertrophy, hemodilution, anemia, retinopathy, neuropathy, nephropathy, hyperlipidemia, hypertension, and increased total body mass have also been described [28–31]. Both the beneficial and collateral effects, such as the TZD effects, seem directly linked to the dose [32]. To minimize the side effects and simultaneously increase the beneficial metabolic effects of AT, the first step of our study was to determine the appropriate PGZ dose. We found that a 5 mg dose over a 26-day period was most efficient for both increasing animal survival and preventing total body mass loss, as compared with larger doses [11,20]. However, no information is available on the collateral effects and/or survival associated with rosiglitazone treatment. In particular, PGZ was used due to fewer reported side-effects and a greater lipogenic action, compared with rosiglitazone [33].

Considering the relevance of assessing early signs of the syndrome for disease outcome in cancer patients, a recent international consensus proposed a definition for cachexia [1], wherein cancer cachexia comprises a continuum of three clinically relevant stages: pre-cachexia (early clinical and metabolic signs), cachexia, and refractory or late-stage cachexia (palliative treatment with a life expectancy less than 3 months). In addition, the importance of early cachexia detection was also addressed (biological signs) and is an important basis for preventive intervention. Thus, in the following discussion, we consider tumor-bearing rats in such a classification scheme.

Another important effect of PGZ treatment is body mass preservation, notably during late-stage of cachexia. Body weight loss is the principal clinical marker of the disease, and a patient in the refractory phase has a life expectancy of approximately 3 months [1,34]. To the best of our knowledge, our work is the first to evaluate the effect of PGZ treatment in solid tumor-bearing rats as a potential approach not only for increasing cachectic animal survival, but also for attenuating the effects of cachexia. Thus, cachexia induced by Walker 256 tumor, after 14 days, induces 10 to 15% of weight loss (from cachexia to the refractory stage). During the same period, TP5 animals lost only 3 to 6% (from pre-cachexia to cachexia) of total body weight. This finding not only demonstrates weight loss attenuation by 40%, but it also demonstrates delayed onset of the disease. However, despite the important clinical relevance suggested by our findings, additional studies must be performed for clarification. In addition, we assessed the impact of PGZ treatment on RPAT, which is the most affected depot during syndrome development [10]. The treatment preserved the RPAT mass, at least in a transitory manner, during the early stages of the disease. Considering the relevance of WAT as one of the first tissues affected by the syndrome and, consequently, its participation in the related metabolic chaos, it is conceivable that transitory preservation may delay the appearance of cachexia syndrome.

Several studies have addressed the effects of TZD on adipose tissue [11,35,36]. In addition to its role in adipocyte biology and adipogenesis, in the cachexia stage, PGZ might contribute to maintaining the adipose mass throughout regulation of the genes related with lipogenesis control. For this aspect, PGZ was effective at restoring, PPAR-γ, CEBP-α, adiponectin, and LPL gene expression for RPAT, at least during the early stage (day 7), and therefore, was responsible for transitory RPAT preservation. We previously demonstrated that these genes are down-regulated early during cachexia development, which begins day 4 after cell inoculation [10]. Therefore, no clear information links transitory RPAT preservation and amelioration of clinical cachexia signs.

Another relevant aspect demonstrated herein is the effect of PGZ on inhibiting tumor mass growth, which is evident in both the cachexia and refractory stages (day 14 to 26). Interestingly, it has also been shown that a higher RPAT mass observed on day 7 is negatively related to tumor growth. In fact, a previous study showed that TZDs exhibit a potential anti-cancerous effect [19]. However, in cachectic tumor-bearing mice, rosiglitazone treatment that was intended to restore peripheral insulin resistance did not affect tumor mass [20]. Thus, the effect seems related to the TZD class and dosage, tumor type and cachexia experimental model.

In cachexia induced by Walker tumors, PGZ induced a delay in the appearance of cachexia signs, with RPAT transient preservation (day 7), and slowed rates of weight loss and tumor mass growth (day 14 and 26); the latter effect being more pronounced. Additionally, improved survival in cachectic rats may be due to weight loss prevention, considering that weight loss is associated with impaired survival in cancer patients. Even considering the short time-course (7 days) of this model, RPAT mass maintenance may be clinically relevant because it may be related with mass preservation, even for a short time, and may improve anticancer therapy responsiveness and tolerability. On the other hand, the magnitude of the tumor mass increase did not correlate with clinical profile signs; thus, a consistent conclusion on a potential interaction between body weight preservation and reduced tumor growth is difficult to be proposed based on the data. Therefore, despite the potential anti-tumor growth effect of PGZ, the evidence for its related mechanisms is limited.

Anorexia is another prevalent symptom in cachexia and one of the criteria for defining differences between cachexia and pre-cachexia. In cachexia induced by the Walker 256 tumor, anorexia is evident from the 10^th^ day after cell inoculation. During the period between the 10^th^ and 14^th^ days, the treatment did not alleviate anorexia. However, in terminal disease, the treatment was effective in alleviating anorexia from the 23^rd^ day. Thus, considering the effect of PGZ on anorexia, it seems that the early effects demonstrated herein were not consistently correlated with anorexia; despite body weight loss attenuation, anorexia did not change. Interestingly, during the late stage, body weight loss attenuation was greater and followed by amelioration of anorexia. However, determining whether this contributes to survival and the potential mechanisms requires further investigation.

### 4.2. Biochemical profile

Hypoglycemia induced by the Walker Tumor 256 has been described in the literature [37], and even though both groups have demonstrated this effect, PGZ treatment partially improved fasting glycemia during cachexia development. This effect on cachexia-induced hypoglycemia was demonstrated in tumor-bearing animals treated with TZDs [11]. In addition, we demonstrate that the treatment exhibits effects on day 7 (early stage). In fact, this benefit has been described as the result of multiple metabolic changes, including an increase in and/or restoration of glucose uptake and metabolism by muscle cells, an increase in fat mass, reduced lipolysis in the tissue, and diminished glucose release by the liver [38,39]. However, additional studies are necessary to assess the potential mechanisms involved in restoring glycaemia and the potential consequences for managing reduced body fat and tumor growth.

A high concentration of triglycerides (TAG) and total cholesterol are the most common lipid abnormalities in patients with insulin resistance, which is also a condition that oncologic patients present and is evident in certain cancer types [40,41], and in animals with tumor-induced cachexia [42]. Cachexia-induced changes in TAG plasma concentration were not reversed with PGZ treatment. In tumor-induced cachexia animal models, dyslipidemia is described from the 8^th^ day after tumor cell inoculation [42]. In this respect, it is worth noting that the treatment did not affect the cachexia-induced TAG level changes. Furthermore, this alteration is not associated with anorexia, which is present in this animal model, typically beginning the 10^th^ day after tumor cells inoculation [10].

Considering that PGZ treatment eventually induces liver toxicity, we measured serum transaminases levels [43] (AST, ALT) and albumin concentration on the 7^th^ and 14^th^ days. Although both enzymes exhibited changes on the 14^th^ day (cachexia stage), with no effect from PGZ, AST concentration was high at both times (days 7 and 14). However, in response to both cachexia and PGZ, ALT concentration did not exhibit relevant changes on day 7 (early stage). Biochemical data analyses demonstrated that the treatment is relatively safe, particularly during the initial stages of cachexia. However, even considering the beneficial effect on fasting glycaemia, hepatotoxicity should be considered when evaluating PGZ treatment as potential anti-cachectic therapeutic approach.

### 4.3. Glucose uptake from isolated adipocytes

Disrupted energy homeostasis seems to be a main target for generating the metabolic “chaos” established during the cachexia syndrome [40,41]. Changes in carbohydrate, lipid and protein metabolism have been described both in cachectic patients [39,44] and tumor-bearing animals [9,10,27]. Glucose metabolism abnormalities have similarly been described, such as glucose intolerance, increased hepatic production (gluconeogenesis) and recycling of glucose carbons, hypoglycemia, hypoinsulinemia and resistance to peripheral insulin resistance [37,39,45]. Furthermore, the 256 Walker tumor is an avid glucose consumer [46]; thus, it is a major source for ATP and lactic acid generation through glycolysis, which stimulates hepatic gluconeogenesis [37]. Despite the importance of glucose in adipose tissue metabolism, as far as we know, this is the first study that has evaluated glucose uptake (^3^H 2-deoxy-D-Glucose) during early-stage cachexia. Considering this aspect, isolated RPAT adipocytes in cachectic rats exhibited lower insulin-stimulated glucose uptake, which was fully restored in the TP5 adipocytes. Our previous studies showed that the RPAT undergoes a profound mass reduction due to cachexia. In addition, its mass decreases the most compared with other visceral adipose tissue depots [10]. Further, the genes related to AT development (PPARγ and CEBPα) were down-regulated on day 4 after tumor cell inoculation (very early stage) [10]. This change may be important to the RPAT capacity to store TAG and, consequently, impair maintenance of its tissue mass.

As previously described, PGZ treatment transiently preserved RPAT mass during early-stage cachexia. In addition, despite the reduced glucose uptake in cachectic animals, the treatment was effective in restoring insulin sensitivity in adipocytes isolated from RPAT. Glucose is a major substrate for TAG formation, because hexose retained by the adipocyte can be incorporated into both the fatty acid (*de novo* lipogenesis) and glycerol molecule moieties of TAG. Additionally, its full oxidation provides energy for maintaining endergonic processes, such as lipogenesis; this effect may contribute to the enhanced RPAT preservation, glucose metabolism maintenance and consequent delay of the initial alterations that establish the syndrome, particularly the alterations that affect fat depots.

In fact, well-described TZD effects include insulin sensitization and the promotion of fatty acid storage by adipose tissue [47]. TZDs promote the appearance of new fat cells that are smaller and more insulin-sensitive, which yield greater insulin-dependent glucose uptake and greater TAG synthesis [13]. Evidence suggests that TZDs, through PPARγ activation, directly modulate the insulin transduction pathway in adipose tissue [48], which regulates expression of various genes involved in glucose metabolism, such as GLUT 1 (constitutive) and GLUT 4 (sensitive to insulin) [47]. However, although PGZ treatment improved glucose uptake in isolated adipocytes from cachectic rats, the mechanisms involved remain unknown.

## Conclusion

The results demonstrate that treatment with 5 mg/kg of PGZ delayed the onset of cachexia clinical signs, followed by an increase in cachectic rat survival, suggesting a potential anti-cachectic effect. PGZ treatment was effective during the early stages of the disease, which was demonstrated by transient adipose tissue mass preservation and insulin-sensitivity restoration in adipocytes. Furthermore, the relationship between greater RPAT mass induced by PGZ and reduced weight loss corroborates the hypothesis that preserving WAT may slow cachexia development. At late-stage cachexia, weight loss and anorexia were attenuated. Finally, in addition to the TZD anti-tumor properties, a potential anti-cachectic effect with eventual potential in the therapeutic treatment of the syndrome is proposed.
